# The Phylogeography of Rabies in Grenada, West Indies, and Implications for Control

**DOI:** 10.1371/journal.pntd.0003251

**Published:** 2014-10-16

**Authors:** Ulrike Zieger, Denise A. Marston, Ravindra Sharma, Alfred Chikweto, Keshaw Tiwari, Muzzamil Sayyid, Bowen Louison, Hooman Goharriz, Katja Voller, Andrew C. Breed, Dirk Werling, Anthony R. Fooks, Daniel L. Horton

**Affiliations:** 1 School of Veterinary Medicine, St. George's University, St. George's, Grenada; 2 Animal Health and Veterinary Laboratories Agency, New Haw, Addlestone, Surrey, United Kingdom; 3 Ministry of Agriculture, St. George's, Grenada; 4 School of Veterinary Science, University of Adelaide, Adelaide, South Australia, Australia; 5 Royal Veterinary College, University of London, Hatfield, United Kingdom; 6 Department of Clinical Infection, Microbiology and Immunology, Institute of Infection and Global Health, University of Liverpool, Liverpool, United Kingdom; 7 School of Veterinary Medicine, University of Surrey, Guilford, United Kingdom; The Global Alliance for Rabies Control, United States of America

## Abstract

In Grenada, West Indies, rabies is endemic, and is thought to be maintained in a wildlife host, the small Indian mongoose (*Herpestes auropunctatus*) with occasional spillover into other hosts. Therefore, the present study was undertaken to improve understanding of rabies epidemiology in Grenada and to inform rabies control policy. Mongooses were trapped island-wide between April 2011 and March 2013 and examined for the presence of Rabies virus (RABV) antigen using the direct fluorescent antibody test (dFAT) and PCR, and for serum neutralizing antibodies (SNA) using the fluorescent antibody virus neutralization test (FAVN). An additional cohort of brain samples from clinical rabies suspects submitted between April 2011 and March 2014 were also investigated for the presence of virus. Two of the 171 (1.7%) live-trapped mongooses were RABV positive by FAT and PCR, and 20 (11.7%) had SNAs. Rabies was diagnosed in 31 of the submitted animals with suspicious clinical signs: 16 mongooses, 12 dogs, 2 cats and 1 goat. Our investigation has revealed that rabies infection spread from the northeast to the southwest of Grenada within the study period. Phylogenetic analysis revealed that the viruses from Grenada formed a monophyletic clade within the cosmopolitan lineage with a common ancestor predicted to have occurred recently (6–23 years ago), and are distinct from those found in Cuba and Puerto Rico, where mongoose rabies is also endemic. These data suggest that it is likely that this specific strain of RABV was imported from European regions rather than the Americas. These data contribute essential information for any potential rabies control program in Grenada and demonstrate the importance of a sound evidence base for planning interventions.

## Author Summary

Rabies, a fatal disease of animals and humans has been endemic in Grenada, West Indies, since the early 1900s. The small Indian mongoose, an introduced animal, is the most likely rabies reservoir, with spillover into domestic animals and humans. To control rabies, large numbers of mongooses were killed in the 1960s/1970s, but this effort did not alter long-term rabies dynamics. Vaccination of dogs, cats and livestock is efficient in protecting these animals, yet is not regularly undertaken. Post-exposure prophylaxis (PEP) in humans is routinely done and no human has died of rabies in Grenada since 1970. However, the threat of rabies and potential to adversely affect the tourism industry, are a burden on the Grenadian government and public. This study has re-evaluated the role of the mongoose in the maintenance of rabies in Grenada, and for the first time, the rabies virus circulating in Grenada has been described. Grenada offers optimal conditions for an oral rabies vaccination (ORV) program, being an island with strict live animal import controls, and a single wildlife rabies reservoir. Although further work is needed before an ORV campaign could be implemented, elimination of rabies from Grenada seems a realistic goal.

## Introduction

Rabies is a globally distributed zoonotic, almost always fatal, infectious disease of the central nervous system of mammals caused by members of the *Lyssavirus* genus. Fourteen *Lyssavirus* species have been classified, including two described recently [1], [2], [3], [4]. The majority of human rabies cases are caused by rabies virus (RABV), transmitted by the bite of infected animals, most commonly dogs and other carnivores [5]. However, rabies caused by lyssaviruses of other species, which are geographically more restricted and circulate mostly in bats, is clinically indistinguishable [6]. Rabies virus strains that circulate in specific hosts or in specific geographic regions are known to undergo genetic adaptations forming distinct biotypes and variants, which can affect their pathogenicity [7], [8]. Rabies occurs worldwide, except in Antarctica. Most human fatalities are seen in Asia, Africa and Latin America, where post-exposure prophylaxis may be unavailable and animal control and vaccination programs are not vigorously enforced [9].

In Grenada, a small island in the Lesser Antilles of the Caribbean, rabies was first suspected in the early 1900s, and was confirmed in the 1950s. It had caused an outbreak in livestock, and the small Indian mongoose (*Herpestes auropunctatus*) was considered the main reservoir host [10].The small Indian mongoose was introduced on 27 Caribbean islands including Grenada in the 1870s to control rats and snakes in sugar cane plantations. Although ineffective at controlling these two pests, the mongoose has become successfully established. It is held responsible for the decline of some of the indigenous wildlife species [11] and is now listed among the world's top 100 worst invasive species [12]. Its greatest public health significance is its role as a disease reservoir for rabies in the Caribbean islands of Cuba, Puerto Rico and Hispaniola [10], [13], [14], [15]. The Grenadian mongoose population is estimated at 200,000, with an average mongoose density of six per hectare [16], [17]. Although mongooses occupy very small home ranges of 2.2 to 4.2 ha [11], rabid mongooses can travel longer distances, and have been recorded 2 km away from their home range [15].

Previous studies have demonstrated separate origins for the rabies viruses that have been detected in the Caribbean. In Puerto Rico, rabies was suspected prior to the introduction of mongooses. Two distinct viral variants, which are closely related to the north central skunk strain, now circulate in two separate locations in Puerto Rico, suggesting two independent introductions of rabies [14]. In Cuba, the rabies strain circulating in terrestrial animals is closely related to the Mexican dog strain [13]. These Caribbean variants are quite different from those circulating in mongooses in Asia [13]. In contrast, on the south Caribbean island of Trinidad, rabies circulates in vampire and other bat species, but rabies has not been reported in the Trinidadian mongoose population [18].

In Grenada, studies were conducted as part of a government rabies control program with the most intense surveillance period from 1968 to 1977 [15], [16], [19], [20]. During these 10 years, 699 cases of rabies were confirmed in animals showing clinical signs of rabies, of which 77% were mongooses [21]. All of the cases of rabies in livestock with a known source and 57% of human rabies exposures treated for post exposure prophylaxis (PEP) (n = 119) were reported to be caused by mongoose bites. In the same 10 year period, close to 12,000 mongooses were trapped and examined for rabies virus antigen using the fluorescent antibody test (FAT) [16]. Mongoose sera were collected from 1971 to 1974 and tested for serum neutralizing antibodies [22]. The authors reported that during these 4 years the antibody prevalence increased while the percentage of rabid mongooses declined, consistent with a trend of declining disease in the mongoose population [21]. Since the 1980s, rabies surveillance in Grenada has been less intense and was further interrupted by Hurricane Ivan in 2004, and Hurricane Emily in 2005. Although the last reported human death due to rabies in Grenada occurred in 1970 [21], rabies remains of great public health concern. According to the Ministry of Health, 60–80 people in Grenada have required PEP annually during recent years as a consequence of mongoose or dog bites. This is a higher rate of PEP treatments than was seen in the 1968 to 1977 period, where 5–45 persons annually required treatment [16]. Rabies vaccination in dogs is inconsistent and dog bites featured second after mongoose bites as the reason for PEP in humans [16]. Travel advice given by the World Health Organization (WHO) for visitors to rabies endemic countries like Grenada includes rabies vaccination [23], which could adversely affect Grenada's tourist industry.

The rabies control policy in Grenada includes import control of live animals, stray animal control, vaccination of domestic animals, and PEP for exposed humans. According to Ministry of Agriculture estimates, 20–25% of the estimated 30,000 dogs on the island are rabies protected at any one time (B. Louison, unpublished data). Grenada is by far the smallest of the rabies endemic Caribbean islands with little import of live animals, and could therefore be an ideal location for a rabies elimination program. The present study was undertaken to evaluate the role of mongooses in the maintenance of rabies in Grenada. Here we present results of systematic and opportunistic surveillance for rabies undertaken over a 24 and 36 month period, respectively, combined with an evolutionary analysis of circulating strains. In addition to providing invaluable information for any potential control policy on Grenada, these data add to our understanding of the evolution and maintenance of an important viral pathogen in a wildlife host.

## Material and Methods

### Ethics statement

All animal work was undertaken according to the American Veterinary Medical Association (AVMA) guidelines on euthanasia (2007). The study was approved by St Georges University (Grenada) Institutional Animal Care and Use Committee (IACUC) on 29 Mar 2010 under IACUC# 10003-R.

### Study location

Grenada is situated approximately 100 km north of Trinidad and just south of the Grenadines. This volcanic island encompasses 344 square kilometers with a human population reported to be 109,100 in July 2012 [24]. Most of the native tropical vegetation has been disturbed by settlements and agriculture, leaving only 9% of the land covered by forests and woodlands. The sugar cane industry, which was the main cash crop during colonial times, collapsed in Grenada in the early twentieth century, and the major agricultural products now are bananas, cocoa, nutmeg, citrus and avocadoes [24]. Livestock production is largely at subsistence level with a few commercial poultry and pig farms.

### Sample collection/active survey

Mongooses were trapped island-wide between April 2011 and March 2013, focusing on locations where mongoose densities were known to be high. Cat live traps were used (65 cm L×18 cm W×18 cm H; Tomahawk, Hazelhurst, WI, USA) baited with chicken or fish parts. Traps were set in shady areas before dawn, collected by midday at the latest and transported to SGU-SVM for immediate processing. Captured animals were briefly restrained using trap dividers and anaesthetized via intra-muscular injection of ketamine (10 mg kg^−1^ body weight; Ketamine hydrochloride, Rotexmedica®, Trittau, Germany) and xylazine (0.2 mg kg^−1^ body weight; AnaSed®, Decatur, IL, USA). Blood was collected via cardiac puncture, and sera were stored at −80°C. Animals were euthanized via intra-cardiac injection of potassium chloride (1–2 mmol kg^−1^ body weight; KCl, Fair Lawn, NJ, USA). To sample saliva, the animals' oral cavities were flushed with 1.0 ml distilled water and the recovered fluid was stored at −80°C. The animals' brains were then removed and kept at 4°C until testing.

### Sample collection/passive survey

Suspect rabies cases were directly submitted through standard passive surveillance procedures via the SGU Small Animal Hospital, the Animal Control Unit of the Ministry of Health, local veterinarians, farmers and pet owners to the Department of Pathology (School of Veterinary Medicine, St. George's University; SGU-SVM). All cases submitted between April 2011 and March 2014 were used in this study. Submitted animals had shown neurological signs, such as paralysis, behavioral changes and/or aggression. In addition, wild animals found freshly dead on roads and presumably hit by cars were collected. Passive surveillance was assumed to be spatially and temporarily consistent over the study period. Brains and saliva samples were taken as described for the trapped mongooses.

### Rabies antigen detection

Rabies was diagnosed by the detection of viral antigen in brain tissue using the direct fluorescent antibody test (FAT) [25]. Separate impression smears of cross sections of the brain stem and the cerebellum were made on 2-ringed slides within two hours of collection. Where brain structures were unrecognizable due to head trauma or maceration, material from two separate areas was used. Smears were fixed in cold acetone and stained with a cocktail of three fluorescein-labeled monoclonal antibodies directed against the rabies nucleocapsid (N) protein (Light Diagnostics™ Rabies DFA reagent, Millipore, Livingston, UK) following suppliers instructions. Slides were viewed under a fluorescence microscope (Nikon Eclipse 80i; D-FL-EPI Fluorescence Attachment; Melville, N.Y., USA) and antigen detection was graded on a scale of 0 to 5 depending on the amount of fluorescent stain, as described previously [26]. Brain material from all inconclusive and rabies positive animals were sent to the OIE Reference Laboratory at the Animal Health and Veterinary Laboratories Agency (AHVLA, UK) for confirmation of diagnosis and virus characterization.

### Molecular diagnostics performed at SGU-SVM

Total RNA was extracted from 30–50 mg pestle-homogenized brain tissue (mixed brain stem and cerebellum), or 0.5 ml saliva samples, using Trizol reagent (Invitrogen, Grand Island, New York, US) according to manufacturer's instructions. The RT-PCR one-step iScript protocol (Biorad Laboratories, Hercules, CA, USA) was used on 200–600 ng total RNA per 50 µl reaction mix. The primers used for amplification targeted a 110 bp fragment of the highly conserved region of the nucleoprotein (N)-gene, and had the following sequence: JW12 5′-ATGTAACACCYCTACAATG-3′ and N165-146 5-′GCAGGGTAYTTRTACTCATA-3′ [27]. The reverse transcription and amplification reactions were performed in an Eppendorf Mastercycler ProS (Eppendorf AG, Hamburg, Germany) and PCR products were visualized using an ethidium bromide-stained 3% agarose gel electrophoresis. In case of a positive RT-PCR, saliva samples from such animals were checked in addition for the presence of RABV by RT-PCR.

### Molecular diagnostics and sequencing performed at AHVLA

Selected brain samples were sent to AHVLA for independent confirmation of results (Table 1). Extracted nucleic acids (Trizol, Invitrogen) were tested by a differential real-time Taqman RT-PCR assay as previously described using the primers JW12, N165-145 and a RABV specific probe [28]. A 606 bp region of the N-gene of positive samples was amplified by hemi-nested RT-PCR [29] and the full nucleoprotein of a subset of isolates (n = 23) was then derived using Sanger sequencing (ABI) with overlapping primers designed from a Grenadian strain (primer sequences available on request). At least one forward and one reverse primer were used to derive consensus sequences, which were then aligned using CLUSTALX.

**Table 1:** Rabies virus positive animals from Grenada from April 2011 to March 2014.

Sample ID	RV No.	Species	Date	Parish	Location	GenBank Accesion No.
***Rabies Suspects***						
**R1**	RV2847	Dog	1 Apr 11	St. Andrew	Birchgrove	KJ957431
**R2**	RV2848	Goat	6 May 11	St. Andrew	MorneLongue	KJ957432
**R4**	RV2849	Dog	15 Jul 11	St. Patrick	Plains	KJ957433
**R5**	RV2850	Cat	6 Sep 11	St. Patrick	Mt Craven	KJ957434
**R6**	RV2851	Dog	26 Sep 11	St. Patrick	Mt Rich	KJ957435
**R7**	RV2852	Dog	29 Sep 11	St. Andrew	Birchgrove	KJ957436
**R8**	RV2853	Mongoose	30 May 11	St. John	Gouyave	KJ957437
**R9**	RV2854	Mongoose	26 Sep 11	St. John	Clozier	KJ957438
**R10**	RV2925	Mongoose	13 Oct 11	St. Andrew	Birchgrove	KJ957440
**R11**	RV2855	Dog	20 Oct 11	St. Andrew	Birchgrove	KJ957439
**R12**	RV2928	Mongoose	10 Jan 12	St. Mark	Victoria	KJ957442
**R13**	RV2929	Dog	5 Mar 12	St. Andrew	Hope	n/a
**R16**	RV2930	Dog	22 May 12	St. Andrew	Birchgrove	n/a
**R18**	RV2964	Mongoose	28 Jun 12	St. David	St. David's	KJ957443
**R23**	RV2965	Mongoose	27 Nov 12	St. John	Gouyave	KJ957444
**R25**	RV2966	Mongoose	10 Dec 12	St. George	Mardigras	KJ957445
**R28**	RV2967	Dog	9 Jan 13	St. George	La Mode	KJ957446
**R29**	RV2968	Dog	17 Jan 13	St. Mark	Waltham	KJ957447
**R31**	RV2969	Dog	14 Feb 13	St. Andrew	Byland	KJ957448
**R33**	RV2970	Mongoose	4 Mar 13	St. George	Grenville Vale	KJ957449
**R34**	RV2971	Mongoose	5 Mar 13	St. George	Vendome	KJ957450
**R36**	RV2972	Mongoose	27 Mar 13	St. Patrick	NonPareil	KJ957451
**R39**	RV2973	Mongoose	16 Apr 13	St. John	Gouyave	KJ957452
**R44**	n/a	Mongoose	13 May 13	St. George	Ft. Jeudy	n/a
**R45**	n/a	Cat	14 May 13	St. John	Gouyave/Maran	n/a
**R46**	n/a	Dog	24 May 13	St. George	Calivigny	n/a
**R49**	n/a	Mongoose	27 May 13	St. George	New Hampshire	n/a
**R53**	n/a	Mongoose	27 Sep 13	St. George	Ft Jeudy	n/a
**R56**	n/a	Mongoose	30 Dec 13	St. John	Mon Plaisir	n/a
**R57**	n/a	Dog	17 Feb 14	St. George	Ft. Jeudy	n/a
**R59**	n/a	Mongoose	27 Feb 14	St. George	Boca	n/a
***Trapped Mongooses***						
**Mg 80**	RV2926	Mongoose	23-Apr-12	St. Mark	NonPareil	KM067274
**Mg 95**	n/a	Mongoose	24-Sep-12	St. George	Mt. Moritz	n/a
***Road Kills***						
**R17**	RV2927	Mongoose	9-May-12	St. Andrew	Balthazar	KJ957441

n/a not available.

### Serological analysis performed at AHVLA

Sera were heat inactivated at 56°C for 30 min, and the presence of rabies virus antibodies was measured using the fluorescent antibody virus neutralization (FAVN) test with a fixed quantity of rabies virus (Challenge Virus Standard (CVS-11)) as previously described [30]. Titers are expressed in IU (International Units) per ml by comparison to a standard serum.

### Evolutionary analysis

Bayesian evolutionary analyses of 23 rabies virus N gene sequences from Grenada were implemented in the BEAST package (v.1.8) [31] with an TN93 substitution model (with gamma distribution of rate variation among sites, and a proportion of invariant sites) selected using the Bayesian Information Criterion in MEGA6 [32]. The 1350 bp N-gene sequences from the Grenada strains were compared to a global panel of 80 rabies viruses (details in Figure S1) using either a relaxed (uncorrelated lognormal) or strict molecular clock and a constant or flexible (Bayesian skyline) population prior. Two chains of 30 million iterations were run for each analysis, combined using LogCombiner (v.1.8.0) with 10% burnin and compared using Tracer (v1.6). Key parameters for each combination of models were compared (Table 2) including an analogue of Akaike's Information Criterion through Markov Chain Monte Carlo simulation (AICM) [33]. Maximum clade credibility trees were chosen using TreeAnnotator, and the resulting trees were visualized using FigTree (v1.4.0). A phylogeny was also inferred using maximum likelihood algorithm, with the same (TN93+G+I) nucleotide substitution model in MEGA6 [32] for comparison with Bayesian analyses.

**Table 2:** Molecular clock model output.

Molecular clock	Population model prior	AICM++	Mean substitution rate (95% HPD)	TMRCA Grenadian strains*	Substitute rate Grenadian branch
Strict	Bayesian skyline	28430	3.26×10^−4^ (2.49–4.04×10^−4^)	16.4 yrs ago (8.6–25.1)	3.26×10^−4^ (2.49–4.04×10^−4^)
Strict	Constant	28440	2.78×10^−4^ (1.96–3.57×10^−4^)	23.4 yrs ago (14.6–34.8)	2.78×10^−4^ (1.96–3.57×10^−4^)
Uncorrelated lognormal	Bayesian skyline	28360	3.99×10^−4^ (2.88–5.15×10^−4^)	11.6 yrs ago (5.5–23.0)	5.09×10^−4^ (2.99–7.40×10^−4^)
Uncorrelated lognormal	Constant	28370	3.17×10^−4^ (2.01–4.33×10^−4^)	22.7 yrs ago (12.6–37.8)	4.36×10^−4^ (2.38–6.94×10^−4^)

* years since most recent strains (2013),

++ AICM is an analogue of Akaike's information criterion estimated through Markov Chain Monte Carlo simulation using the method-of-moments estimator [33]. Lower values indicate better model fit.

## Results

**Figure 1:**
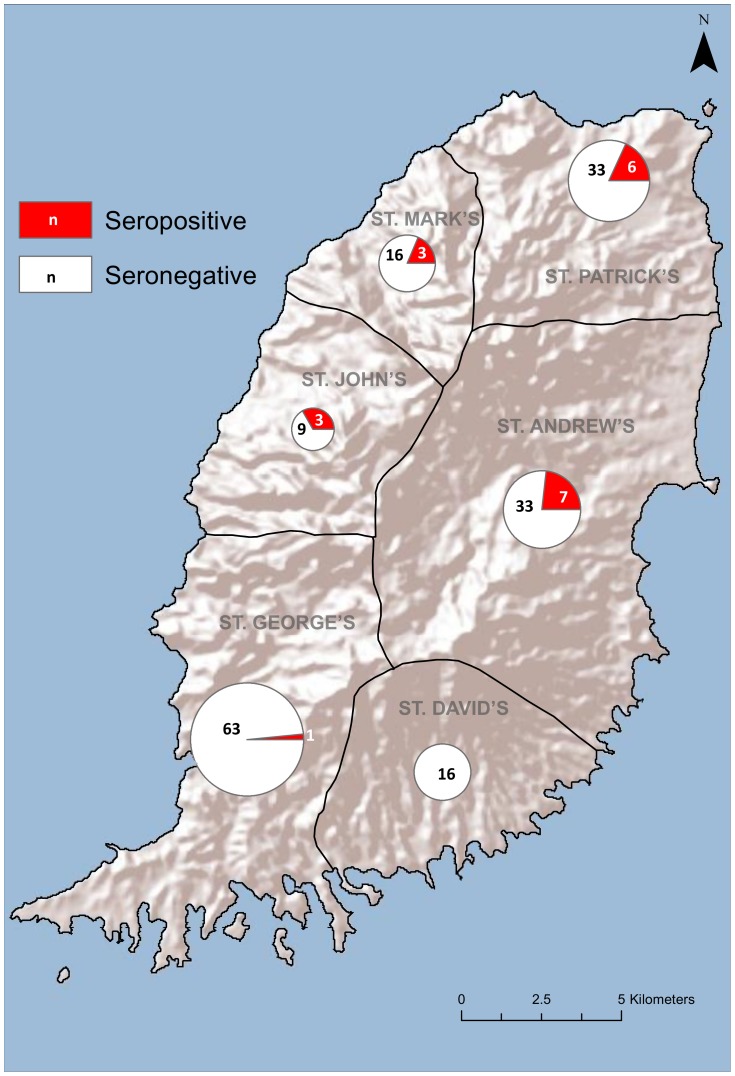
Number and distribution of mongooses trapped per parish in Grenada between Apr 2011 and Mar 2013. The proportions of seropositive mongooses are indicated, with the area of the circle proportional to the total number of animals trapped in each parish. Only one seropositive mongoose was caught during April 2011 and March 2012, in the parish of St Patrick.

A total of 171 mongooses were trapped and sampled in the 24 month period between April 2011 and March 2013. The distribution and number of mongooses trapped in each of Grenada's six parishes is shown in Figure 1. The composition was as follows: 115 adult males and 56 adult females; no juveniles were caught. Mean values for body weight and body length (excluding tail) were: 700 (±120) g and 35.3 (±2.0) cm for adult males; 490 (±84) g and 31.8 (±1.8) cm for adult females. The brains of all 171 animals were tested for RABV antigen and RNA using FAT and RT-PCR at SGU. One mongoose (adult female # 95; St. George parish) was clearly FAT positive, graded 3+. A second mongoose (adult male #80; St. Mark parish) was weakly positive, graded 1+. The saliva samples of both animals were RT-PCR negative using the one-step iScript protocol at SGU, and neither of these two mongooses had shown any abnormal behavior while in their traps. RT-PCR at SGU, real time-RT-PCR at AHVLA and hnRT-PCR detected rabies virus RNA in brain samples of these two mongooses. All remaining 169 mongooses were negative by FAT and RT-PCR. Serology investigations of the live-caught mongooses revealed 20 of 171 (11.7%, 95% CI 6.9–16.5%) had rabies neutralizing antibodies above the standard threshold of 0.5 IU/ml. This proportion is lower than the lower limit seroprevalence estimates in previous studies, where 31 out of 149 mongoose were seropositive (20.8%, 95% CI 14.3% to 27.3%) (Fischer's exact test, p = 0.03) [22]. However, using a less conservative threshold of >0.1 IU, 33 of 171 mongooses in this study (19.3%, 95% CI 13.4–25.2%) would be considered seropositive (titer range 0.1 to 3.42 IU ml^−1^), which is not significantly different from previous estimates (p = 0.9). All 20 seropositive mongooses came from the northern and central areas of Grenada.

**Figure 2:**
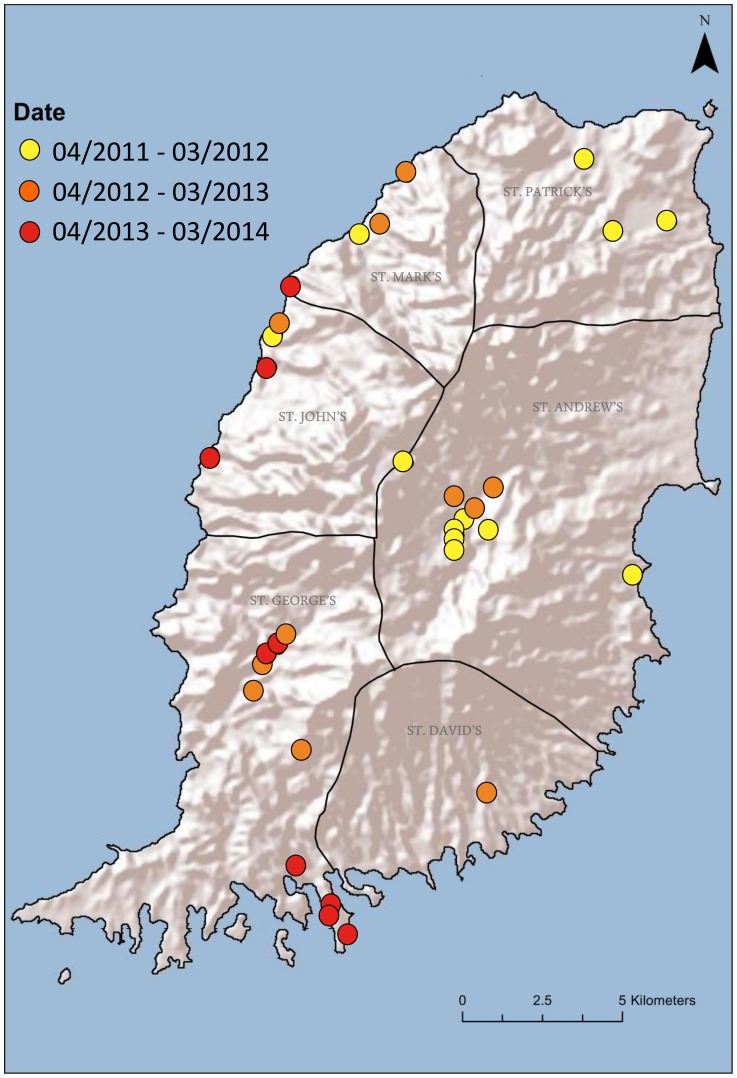
Distribution of rabies cases in Grenada in period 1 (Apr 11–Mar 12), period 2 (Apr 12–Mar 13) and period 3 (Apr 13–Mar 14).

Between April 2011 and March 2014 (36 months), 48 animals were submitted as rabies suspects to SGU-SVM: 26 dogs, 16 mongooses, 3 cats and 3 goats. All submitted mongooses were killed during attacks on humans or dogs; bite exposure of other domestic animals very likely occurred, but were not seen or reported. Most dogs and cats had shown aggression and bitten humans or domestic animals, some had shown signs of paralysis without aggressive behavior, and the goats had shown behavioral changes consistent with neurological disease. Rabies was diagnosed in 31 of these 48 cases: 23 were verified by FAT, RT-PCR and sequencing and a further 8 animals by FAT and RT-PCR only at SGU. These 31 cases consisted of 16 mongooses, 12 dogs, 2 cats and 1 goat (Table 1). A subset of these positive animals had saliva samples taken for molecular testing. Of these samples, 100% (12/12) mongooses, 100% cats (1/1) and 60% dogs (3/5) had RABV detectable by RT-PCR in saliva. The distribution of rabies cases in Grenada is shown in Figure 2. Rabies cases were detected in all parishes with a cluster in the Birchgrove area (parish of St. Andrew). All rabies cases recorded between April 2011 and March 2012 came from the northern and central parts of the island. Since June 2012, rabies cases were distributed throughout the island with the majority seen in the southern and western parts of the island, suggesting of spread of disease across the island from North-East to South-West, which is supported by a correlation between latitude and date of case submission (r = −0.63, p = 0.0001, Figure S2). There were 21 rabies cases during the dry seasons from December to May and only 10 rabies cases during the wet seasons from June to November (Table 1).

Road kills: During the study period, eight animals (three mongooses, three common opossums (*Didelphis marsupialis*) and two leaf-nosed bats (*Carollia perspicillata*)) were found dead and were examined. One mongoose (R17), a pregnant female, was RABV positive, diagnosed by FAT, RT-PCR and sequencing (Table 1).

**Figure 3:**
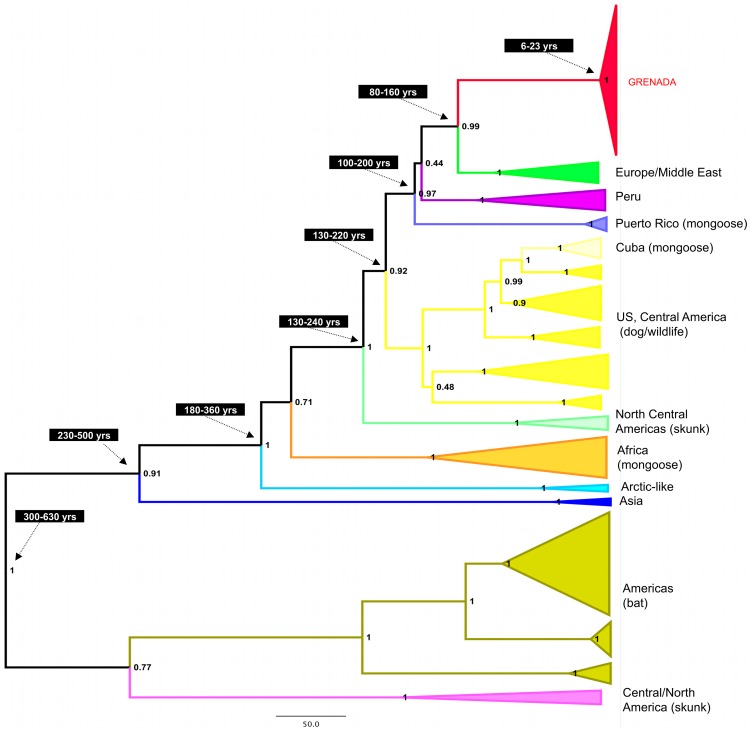
A maximum clade credibility tree, comparing 1350 bp N-gene sequence of rabies virus strains from Grenada (n = 23) with representatives from a global panel of 80 rabies viruses (reference sequence identities in Figure S2). A relaxed (uncorrelated lognormal) molecular clock and Bayesian skyline population prior were used for 60 million iterations in the BEAST package (v1.8) The posterior probability is indicated at each node and estimates of the dates for ancestral nodes are indicated. Scale bar represents 50 years.

To assess the relationship of the Grenadian RABV to other RABV, the 23 viral sequences generated from Grenadian isolates were analyzed for this study (Figures 3, S2 and S3). The full N-gene Bayesian analysis allowed robust inference of the evolutionary relationships among a global panel of rabies viruses and allowed estimation of the dates of the most recent common ancestors, as shown in Figure 3. The best supported molecular clock model was a relaxed (uncorrelated lognormal) model with a Bayesian skyline population prior. The viruses from Grenada form a strongly supported monophyletic clade with a common ancestor predicted by these data to have occurred very recently (95% HPD 6–23 years ago with the best fit model). Estimates of the time to the most recent common ancestor (TMRCA) for the Grenadian strains were consistently recent with all models and although variable (median TMRCA ranged from 11.5 to 23.4 years) the estimates were not significantly different and the oldest estimated date was 40 years ago (with a constant population prior) (Table 2). For both analyses with relaxed molecular clocks, the branch rate in the Grenadian tree was higher than the mean substitution rate across the whole tree, but not significantly so. The most closely related viruses to the Grenadian strains in this data set form another well supported clade of viruses from Europe and the Middle East, which shares a common ancestor with viruses from Grenada, predicted to have occurred 83 to 161 years ago. Samples from mongooses in Cuba and Puerto Rico are in separate and distinct clades, but all are in the ‘cosmopolitan’ lineage of rabies viruses. Bat associated RABVs from South and Central America have a separate origin, and there is no evidence from these data that rabies in Grenada is caused by known bat variants. Tree topology using the maximum likelihood method was similar, with rabies viruses from mongoose in Grenada, Puerto Rico and Cuba, all in distinct lineages (Figure S3).

## Discussion

Rabies is endemic in Grenada, yet the most recent previous rabies surveillance was conducted in the 1960s/70s. Key for any rabies control strategy is access to up-to-date information about the infection dynamics and circulating virus strains. The current study with its active and passive rabies surveillance program has confirmed that rabies remains endemic in Grenada and is most likely maintained in the small Indian mongoose as the only wildlife host. Among the trapped mongooses, we found 2 of 171 animals positive for RABV, giving a RABV proportion positive of 1.7% (95% CI 0–2.78%) over the two years of the study. Despite a difference in sample size, this figure is comparable to studies conducted in the 1970s using a comparable trapping technique [16], where attempts were made to control mongoose numbers by trapping: nearly 12,000 mongooses were culled over a 10 year period and the annual proportion of trapped mongooses found rabid was 2.0% with a range of 0.14% to 3.5%.

There is potential for trapping bias to confound an accurate assessment of the true rabies infection pattern in the mongoose population. Areas with high mongoose densities were preferentially chosen as trapping sites, which might affect capture success of rabid animals. Also, mongooses affected by rabies may not enter traps readily, or conversely may be more likely to encounter and enter traps due to increased roaming. Nevertheless, a proportion of rabid animals among the live-trapped mongooses, combined with the large mongoose population (estimated at close to 200,000 animals on the island) [16], [17] and propensity of rabid mongooses to travel long distances [15] means a substantial proportion of the human and domestic animal population in Grenada are potentially at risk of rabies exposure.

Overall seroprevalence estimates for rabies SNAs in the Grenadian mongoose during the study period, using a conservative threshold, were lower than previously reported [22]. However, using a less conservative threshold of >0.1 IU/ml, which is comparable to the cut-off used in the previous study, the overall seroprevalence is not significantly different to 1970s/1980s estimates. In these studies, Everard et al. [22] found SNAs in 29.7% of mongooses with a range of 20.8% (95% CI 14.3% to 27.3%) in 1971 to 43.2% in 1974 (95%CI 35.6% to 50.8%) using a rabies fluorescent focus inhibition test [95% confidence intervals calculated from samples sizes].

A clear temporal and spatial pattern was observed for the rabies cases seen in the passive surveillance program. During the first year, all rabies cases originated from the northern and central regions. Since the second year, all rabies cases came from the central and south-western regions of Grenada. Whether this indicates an epizootic wave moving from the north-east to the south-west or two independent outbreaks cannot be ascertained without further studies. Sampling bias in the trapped animals precludes confirmation of a temporal pattern in the serology data, but none of the mongooses caught in the southern parts of Grenada had detectable SNAs during these two years. It appears that rabies in Grenada might follow a pattern of epidemic cycles in mongoose, as described elsewhere in the epidemiology of wildlife rabies, such as raccoon rabies in the USA [34] or red fox rabies in Europe [35]. This epidemic pattern could be confirmed by a longitudinal study, and would inform the timing and scale of a successful control program. A seasonal effect was observed: Grenada has only two seasons, dry and wet, and the number of rabies cases during the dry seasons (December to May) was twice as high as during the wet seasons. May is the month with the highest number of cases (n = 7). It is possible that mongooses roam over longer distances due to the lower availability of food during the drier months, facilitating contact with other animals. Mongooses breed throughout the year with 2–3 litters per female; however, maximum number of births occur prior to the summer solstice in June. This would be preceded by maximum breeding activity in April and May, with increased movements and close contact between mongooses [11].

Among the rabies suspects submitted for diagnosis, 16 were mongooses. Healthy mongooses are naturally very shy and avoid human contact. These 16 animals were killed during attacks on either humans or dogs, and it is not surprising that all were rabies positive. Dogs represented the highest suspect case number, and out of 26 submissions, 12 were positive. Two of these dogs had a history of being bitten by mongooses. One dog had a history of a single rabies vaccination within the past 2–3 years, but the vaccinations had not been kept up to date. Most dogs in Grenada are owned, yet many are free roaming, and are not vaccinated regularly. The number of animals submitted for diagnosis is likely to be an under estimate of rabies cases in Grenada. The general public, especially farmers, believe that an aggressive mongoose is highly likely to be rabid and therefore mongooses may be killed without being tested. Exposed domestic animals are typically vaccinated immediately after bites occur.

The number of wild animals found dead or submitted after road traffic accidents was very low (3 common opossums, 3 mongooses, 2 bats), and of these, only one mongoose was diagnosed with rabies. Grenada has very few wild terrestrial mammal species and, except for mongooses, rats and mice, they occur in low numbers [36]: common opossum, mouse opossum (*Marmosa murina*), nine-banded armadillo (*Dasypus novemcinctus*), and Mona monkey (*Cercopithecus mona*). Opossums are not considered susceptible to rabies, yet one was reported rabies positive in a previous study [15]. Rabies has never been reported in a Mona monkey in Grenada.

The vampire bat (*Desmodus rotundus*), which is the main rabies reservoir on the neighboring island of Trinidad and several Latin American countries does not occur on Grenada [18], [36]. Other bat species are lyssavirus reservoirs worldwide [37] and 13 species of insectivorous and fructivorous bats have been recorded in Grenada [38]. A bat survey was conducted in 1973/74 after one Mexican fruit bat (*Artebeus jamaicensis*) with suspicious signs had been confirmed rabid: no virus was isolated from 411 bats tested, but seroprevalence was 40.6% [38]. It is not known if bats transmit rabies to other animals in Grenada. The absence of bat rabies variants among the viruses obtained in this study makes it less likely, but does not rule it out and this would need to be a consideration if rabies elimination was attempted in Grenada. In Trinidad, rabies is transmitted by vampire and other bat species, but is not maintained in the Trinidadian mongoose population [18], which has a low average mongoose density of 2.5 per hectare compared to 6.6 per hectare in Grenada [17].

All the RABV sequences from Grenada are highly genetically related (98.3–100%) and form a distinct clade within the cosmopolitan RABV lineage. Sequences of viruses detected in mongoose had the highest diversity, and sequences from dogs and other domestic animals were distributed throughout the Grenadian clade. These findings do not preclude the possibility of sustained dog to dog transmission, but concur with previous reports and epidemiological data that mongoose are the primary reservoir for rabies in Grenada [21]. The Grenadian strains share an ancestor with a clade of European strains, estimated to have occurred 80–160 years ago, corresponding to the time of the first reports of rabies in Grenada. However, the TMRCA of the Grenadian viruses alone is estimated to have occurred much more recently, within the last 5–40 years (when including 95% HPD estimates from all models). Although care must be taken interpreting estimates of divergence using recently detected similar sequences, these estimates are consistent using both strict and relaxed molecular clocks, and also constant and flexible (Bayesian skyline) population models (Table 2). There are at least three possible explanations for this unexpectedly recent ancestry of the viruses currently circulating in Grenada. Perhaps the most likely explanation is that the rabies virus population in Grenada has undergone a population bottleneck in the recent past, and this bottleneck could coincide with the last recorded period of intensive mongoose rabies control activities in the 1970s and 80s [21]. Natural disasters such as Hurricane Ivan and Emily in 2004 and 2005 are likely to have had an impact on mongoose populations and therefore on virus transmission, although this is hard to assess. Two less likely scenarios are a recent introduction to the island from an unknown source, and the presence of other viruses in Grenada which were not sampled in this study. These seem less likely as potential sources of introduction from other nearby countries are well represented in the phylogenetic analysis, and there was thorough geographic coverage of sampling in Grenada. An additional consideration, particularly for analyses of genes such as the rabies virus N gene, is the potential effect of strong purifying selection masking evolutionary rates. Nucleotide substitution models are unable to properly account for variability in selection pressures, leading to gross underestimates of the origins of viral clades [39]. This underestimation is more likely however, to effect long internal branches than recent ones. Although a significant issue for understanding the ancient origins of viruses such as rabies, its significance here is unclear.

The viral strain in Grenada is most closely related to variants circulating in the middle-eastern and European countries within the cosmopolitan lineage (a world-wide lineage believed to have originated in the old-world and spread across the globe with human movement during the 18^th^ century). These phylogenetic data and other circumstantial evidence suggest that rabies was introduced independently of mongoose introduction: The RABV variants described in Cuba and Puerto Rico are quite different from each other and are different from the Grenadian variant: in Cuba it is closely related to the Mexican dog strain, whereas the two Puerto Rican strains are related to the north central skunk strain that circulates in the US [13], [14]. Mongooses were introduced into Grenada some 35 years before the first rabies suspicion was raised in the early 1900s and although they were released into 27 Caribbean islands, mongoose rabies has only been reported in Cuba, Hispaniola, Puerto Rico and Grenada.

These data have confirmed that rabies persists in Grenada and is likely to be maintained in a wildlife host. Protection of domestic animals and humans is necessary through pre or post exposure prophylaxis, but unless infection in the reservoir is controlled, the public and animal health risks posed by rabies are unlikely to diminish [40]. An understanding of the dynamics of infection in the host is necessary for adequate control. The temporal and geographic variation in rabies infection seen here, alongside genetic evidence for viral population bottleneck suggests epidemic cycles of rabies in the mongoose population. Such cycles have been described for other rabies reservoirs on varying timescales [41], [42], [43], [44]. This cyclic nature would also fit with a report by Everard *et al.* [20]), who found significant fluctuations in the proportion of rabid mongooses between years, indicating a 3–4 years cycle on Grenada. Rabies control by culling or poisoning of mongooses only showed short-lived effects [20]. Oral rabies vaccination (ORV) of other wild animals has been successful in many regions, and vaccination trials with commercially available vaccines under experimental conditions showed that mongooses readily seroconverted [22], [45]. A mongoose specific bait containing an oral vaccine based on genetically modified rabies virus constructs is being developed and has shown promising first results under experimental conditions [46]. The long term economic benefit of an ORV program is difficult to predict due to the highly variable dynamics of rabies in different wildlife hosts and the lack of objectively quantifiable measures of the rabies-related costs [47]. To estimate the expense of an ORV program for Grenada, data such as mongoose densities and distribution, animal movements, bait uptake, and longevity of vaccine-induced immunity should ideally be available. The costs of the current rabies control and prevention program during enzootic and epidemic phases need to be determined, but will include costs related to pet animal and livestock vaccinations, human pre- and post-exposure prophylaxis, survey work, diagnosis and educational material [48]. The long term benefit of reducing these ongoing costs is likely to exceed the short term cost of a successful ORV program. Grenada offers ideal conditions to attempt an ORV campaign: it is a small island, and these data suggest there is a single RABV strain maintained in one host only. Further work quantifying the epidemiology of rabies in mongoose could inform optimized vaccine campaigns to eliminate rabies in this wildlife host. If combined with vaccination of dogs and responsible pet ownership, elimination of rabies in Grenada is a realistic goal.

## Supporting Information

Figure S1 The same maximum clade credibility tree illustrated in Figure 3, except with sequence details for reference strains displayed.
(PDF)

Figure S2 Relationship between latitude and date of submission of rabies cases. There is a significant correlation between latitude of case origin (in decimal degrees) and date of case submission (Pearson's correlation coefficient of −0.63 [95%CI −0.80 to −0.35, p = 0.0001]). These data suggest a temporo-spatial spread from North to South.
(PDF)

Figure S3 Maximum likelihood phylogenetic tree, comparing the same 1350 bp N-gene sequences as in Figures 3 and S1 using the same TN93 nucleotide substitution model with rate variation among sites and a proportion of invariant sites. Bootstrap values (percentage of 100 replicates) are given at significant nodes.
(PDF)

## References

[1] Dietzgen R, Calisher CH, Kurath G, Kuzmin IV, Rodriguez LL, et al.. (2011) Rhabdoviridae. In: Kind A, Adams MJ, Carstens EB, Lefkowitz EJ, editors. Virus Taxonomy; Ninth Report of the International Committee on the Taxonomy of Viruses. San Diego: Elsevier. pp. 654–681.

[2] Kuzmin IV, Mayer AE, Niezgoda M, Markotter W, Agwanda B, et al (2010) Shimoni bat virus, a new representative of the Lyssavirus genus. Virus Res 149: 197–210. 20138934 10.1016/j.virusres.2010.01.018

[3] Freuling CM, Beer M, Conraths FJ, Finke S, Hoffman B, et al (2011) Novel lyssavirus in Natterer's bat, Germany. Emerg Infect Dis 17: 1519–1522. 21801640 10.3201/eid1708.110201 PMC3381583

[4] Marston DA, Horton DL, Ngeleja C, Hampson K, McElhinny LM, et al (2012) Ikoma Lyssavirus, highly divergent novel Lyssavirus in an African civet. Emerg Infect Dis 18: 664–667. 22469151 10.3201/eid1804.111553 PMC3309678

[5] Charlton KM (1988) The pathogenesis of rabies. In: Campbell JM, Charlton KM, editors. Rabies. Boston: Kluwer Academic Publishers. pp. 101–150

[6] Fooks AR, McElhinney LM, Pounder DJ, Finnegan CJ, Mansfield K, et al (2003) Case report: isolation of a European bat lyssavirus type 2a from a fatal human case of rabies encephalitis. J Med Virol 71: 281–9. 12938204 10.1002/jmv.10481

[7] Tuffereau C, Leblois H, Benejan J, Coulon P, Lafay F, et al (1989) Arginine or lysine in position 333 of ERA or CVS glycoprotein is necessary for rabies virulence in adult mice. Virology 172: 206–212. 2505450 10.1016/0042-6822(89)90122-0

[8] Wunner WH, Larson JK, Dietzschold B, Smith CL (1988) The molecular biology of rabies viruses. Rev Infect Dis 10: S771–S784. 2462742 10.1093/clinids/10.supplement_4.s771

[9] Knobel DI, Cleaveland S, Coleman PG, Fevre EM, Meltzer MI, et al (2005) Re-evaluating the burden of rabies in Africa and Asia. Bull World Health Organ 83: 360–368. 15976877 PMC2626230

[10] Jonkers AH, Alexis F, Loregnard R (1969) Mongoose rabies in Grenada. West Indian Med J 18: 167–170. 4918501

[11] Nellis WD (1989) Herpestes auropunctatus. J Mammal 342: 1–6. 4855184

[12] International Union for Conservation of Nature (IUCN)-Global Invasive Species Database (2013) 100 of the world's worst invasive alien species. Available: www.issg.org/publications.htm; accessed 28 May 2013.

[13] Nadin-Davis SA, Torres G, De Los Angeles Ribas M, Guzman M, Cruz De La Paz R, et al (2006) A molecular epidemiological study of rabies in Cuba. Epidemiol Infect 134: 1313–1324. 16740188 10.1017/S0950268806006297 PMC2870515

[14] Nadin-Davis SA, Velez J, Malaga C, Wandeler AI (2008) A molecular epidemiological study of rabies in Puerto Rico. Virus Res 131: 8–15. 17869366 10.1016/j.virusres.2007.08.002

[15] Everard COR, Baer GM, James A (1974) Epidemiology of mongoose rabies in Grenada. J Wildl Dis 10: 190–196. 4602354 10.7589/0090-3558-10.3.190

[16] Everard COR, James AC, DaBreo S (1979) Ten years of rabies surveillance in Grenada. Bull Pan Am Health Organ 13 4:342–353. 526674

[17] Horst RG, Hoagland DB, Kilpatrick WC (2001) The mongoose in the West Indies: the biogeography and population biology of an introduced species. Biography of the West Indies: patterns and perspectives 2: 409–424.

[18] Seetahal JF, Velasco-Villa A, Allicock OM, Adesiyun AA, Bissessar J, et al (2013) Evolutionary history and phylogeography of rabies viruses associated with outbreaks in Trinidad. PLoS Negl Trop Dis 7: e2365. 23991230 10.1371/journal.pntd.0002365 PMC3749974

[19] Everard COR, Murray D, Gilber PK (1972) Rabies in Grenada. Trans R Soc Trop Med Hyg 66 6:878–888 10.1016/0035-9203(72)90123-X 4675867

[20] Everard COR, Everard JD (1992) Mongoose rabies in the Caribbean. Ann N Y Acad Sci 653: 356–366. 1626884 10.1111/j.1749-6632.1992.tb19662.x

[21] Everard COR, Everard JD (1988) Mongoose rabies. Rev Infect Dis 10: S610–614. 3060954 10.1093/clinids/10.supplement_4.s610

[22] Everard COR, Baer GM, Alls ME, Moore SA (1981) Rabies serum neutralizing antibody in mongooses from Grenada. Trans R Soc Trop Med Hyg 75: 654–666. 7330920 10.1016/0035-9203(81)90143-7

[23] World Health Organization (WHO) Media center (2013) Rabies fact sheet. Available: http://www.who.int/mediacentre/factsheets/fs099/en/. Accessed 27 March 2014.

[24] Central Intelligence Agency (CIA) (2013) World Factbook, Grenada. Available: https://www.cia.gov/library/publications/the-world-factbook/geos/gj.html. Accessed April 25, 2013

[25] Dean DJ, Abelseth MK, Atanasiu P (1996) The fluorescent antibody test. In: Meslin FX, Kaplan MM, Koprowski H, editors. Laboratory techniques in rabies, 4th ed. Geneva: World Health Organization. pp. 66–79.

[26] Bingham J, Van Der Merwe M (2002) Distribution of rabies antigen in infected brain material: determining the reliability of different regions of the brain for the rabies fluorescent antibody test. J Virol Methods 101: 85–94. 11849687 10.1016/s0166-0934(01)00423-2

[27] Hayman DT, Banyard AC, Wakeley PR, Harkess G, Marston D, et al (2011) A universal real-time assay for the detection of Lyssaviruses. J Virol Methods 177: 87–93. 21777619 10.1016/j.jviromet.2011.07.002 PMC3191275

[28] Wakeley PR, Johnson N, McElhinney LM, Marston D, Sawyer J, et al (2005) Development of a real-time, TaqMan reverse transcription-PCR assay for detection and differentiation of lyssavirus genotypes 1, 5, and 6. J Clin Microbiol 43: 2786–279. 15956398 10.1128/JCM.43.6.2786-2792.2005 PMC1151886

[29] Heaton RP, Johnstone P, McElhinney LM, Cowley R, O'Sullivan E, et al (1996) Heminested PCR assay for detection of six genotypes of rabies and rabies-related viruses. J Clin Microbiol 35 11:2762–2766. 10.1128/jcm.35.11.2762-2766.1997 PMC230057 9350729

[30] Cliquet F, Aubert M, Sagne L (1998) Development of a fluorescent antibody virus neutralization test (FAVN test) for the quantitation of rabies-neutralizing antibody. J Immunol Methods 212: 79–87. 9671155 10.1016/s0022-1759(97)00212-3

[31] Drummond AJ, Rambaut A (2007) “BEAST: Bayesian evolutionary analysis by sampling trees.”. BMC Evol Biol 7: 214 10.1186/1471-2148-7-214 17996036 PMC2247476

[32] Tamura K, Stecher G, Peterson D, Filipski A, Kumar S (2013) MEGA6: Molecular Evolutionary Genetics Analysis version 6.0. Mol Biol Evol 30: 2725–9. 24132122 10.1093/molbev/mst197 PMC3840312

[33] Baele G, Li WL, Drummond AJ, Suchard MA, Lemey P (2013) Accurate model selection of relaxed molecular clocks in bayesian phylogenetics. Mol Biol Evol 30: 239–43. 23090976 10.1093/molbev/mss243 PMC3548314

[34] Childs JE, Curns AT, Dey ME, Real AL, Rupprecht CE, et al (2001) Rabies epizootics among raccoons vary along a North-South gradient in the Eastern United States. Vector Borne Zoonotic Dis 1: 253–267. 12653126 10.1089/15303660160025895

[35] Wandeler A (2004) Epidemiology and ecology of fox rabies in Europe. In: King AA, Fooks AR, Aubert M, Wandeler AI, editors. Historical perspective of rabies in Europe and the Mediterranean Basin. Paris: World Organisation for Animal Health (OIE). pp. 201–214.

[36] Groome JR (1970) A natural history of the island of Grenada. Trinidad: W.I. Caribbean Printers Ltd. pp 53–61.

[37] Rupprecht CE, Hanlon CA, Hemachudha T (2002) Rabies re-examined. Lancet Infect Dis 2: 327–343. 12144896 10.1016/s1473-3099(02)00287-6

[38] Price JL, Everard COR (1977) Rabies virus and antibody in bats in Grenada and Trinidad. J Wildl Dis 13: 131–135. 864845 10.7589/0090-3558-13.2.131

[39] Wertheim JO, Kosakovsky Pond SL (2011) Purifying selection can obscure the ancient age of viral lineages. Mol Biol Evol 28: 3355–3365 10.1093/molbev/msr170 21705379 PMC3247791

[40] Fooks AR, Banyard AC, Horton DL, Johnson N, McElhinney LM, et al (2014) Current status of rabies and prospects for elimination. Lancet 10.1016/S0140-6736(13)62707-5 PMC7159301 24828901

[41] McLean RG (1975) Raccoon rabies. In: The natural history of rabies, Vol 2. Baer GM, editor. New York & London: Academic Press. pp53–77.

[42] George DB, Webb CT, Farnsworth ML, O'Shea TJ, Bowen RA, et al (2011) Host and viral ecology determine bat rabies seasonality and maintenance. Proc Natl Acad Sci USA 108: 10208–10213. 21646516 10.1073/pnas.1010875108 PMC3121824

[43] Rhodes CJ, Atkinson RP, Anderson RM, Macdonald DW (1998) Rabies in Zimbabwe: reservoir dogs and the implications for disease control. Philos Trans R Soc Lond B Biol Sci 353: 999–1010. 9684293 10.1098/rstb.1998.0263 PMC1692299

[44] Amengual B, Bourhy H, Lopez-Roig M, Serra-Cobo J (2007) Temporal dynamics of European bat Lyssavirus Type 1 and survival of *Myotis myotis* bats in natural colonies. PLoS ONE 2: e566. 17593965 10.1371/journal.pone.0000566 PMC1892799

[45] Blanton JD, Meadows A, Murphy SM, Manangan J, Hanlon CA, et al (2006) Vaccination of small Indian mongoose (*Herpestes javanicus*) against rabies. J Wildl Dis 42: 663–666. 17092899 10.7589/0090-3558-42.3.663

[46] Vos A, Kretzschmar A, Ortmann S, Lojkic I, Habla C, et al (2013) Oral vaccination of captive small Indian mongoose (*Herpestes auropunctatus*) against rabies. J Wildl Dis 49: 1033–1036. 24502736 10.7589/2013-02-035

[47] Gordon ER, Krebs JW, Rupprecht CR, Real LA, Childs JE (2005) Persistence of elevated rabies prevention costs following post-epizootic declines in rates of rabies among raccoons (*Procyon lotor*). Prev Vet Med 68: 195–222. 15820116 10.1016/j.prevetmed.2004.12.007

[48] Sterner RT, Meltzer MI, Shwiff SA, Slate D (2009) Tactics and economics of wildlife oral rabies vaccination, Canada and the United States. Emerg Infect Dis 15: 1176–1184 10.3201/eid1508.081061 19757549 PMC2815952

