# Supplementary figures and images for: The Phylogeography of Rabies in Grenada, West Indies, and Implications for Control
Source: PLoS Negl Trop Dis. 2014 Oct 16;8(10):e3251. doi: 10.1371/journal.pntd.0003251 (PMC4199513; doi:10.1371/journal.pntd.0003251)

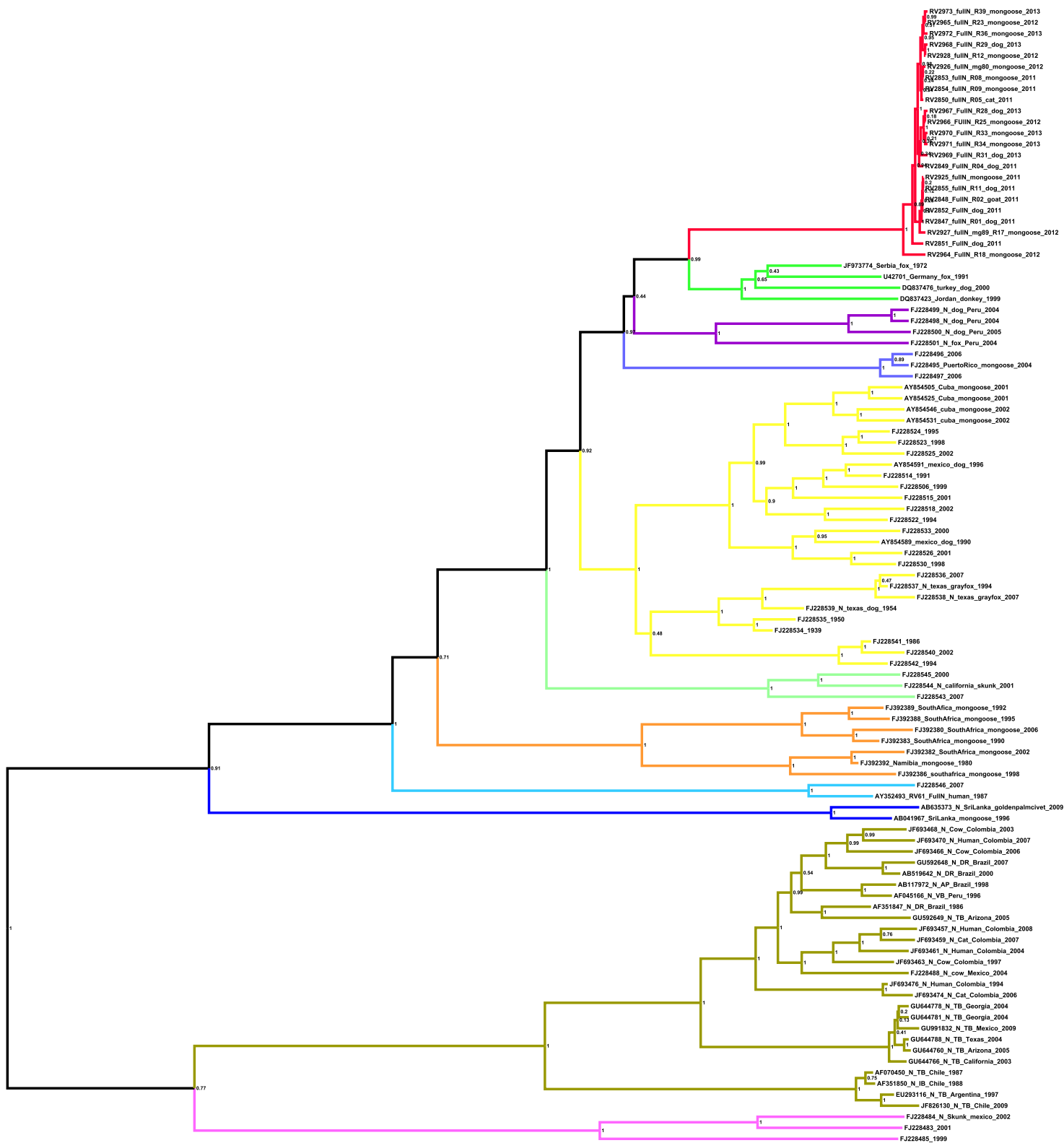

Supplement: Figure S1 — The same maximum clade credibility tree illustrated in Figure 3, except with sequence details for reference strains displayed. (PDF) [file pntd.0003251.s001.pdf]

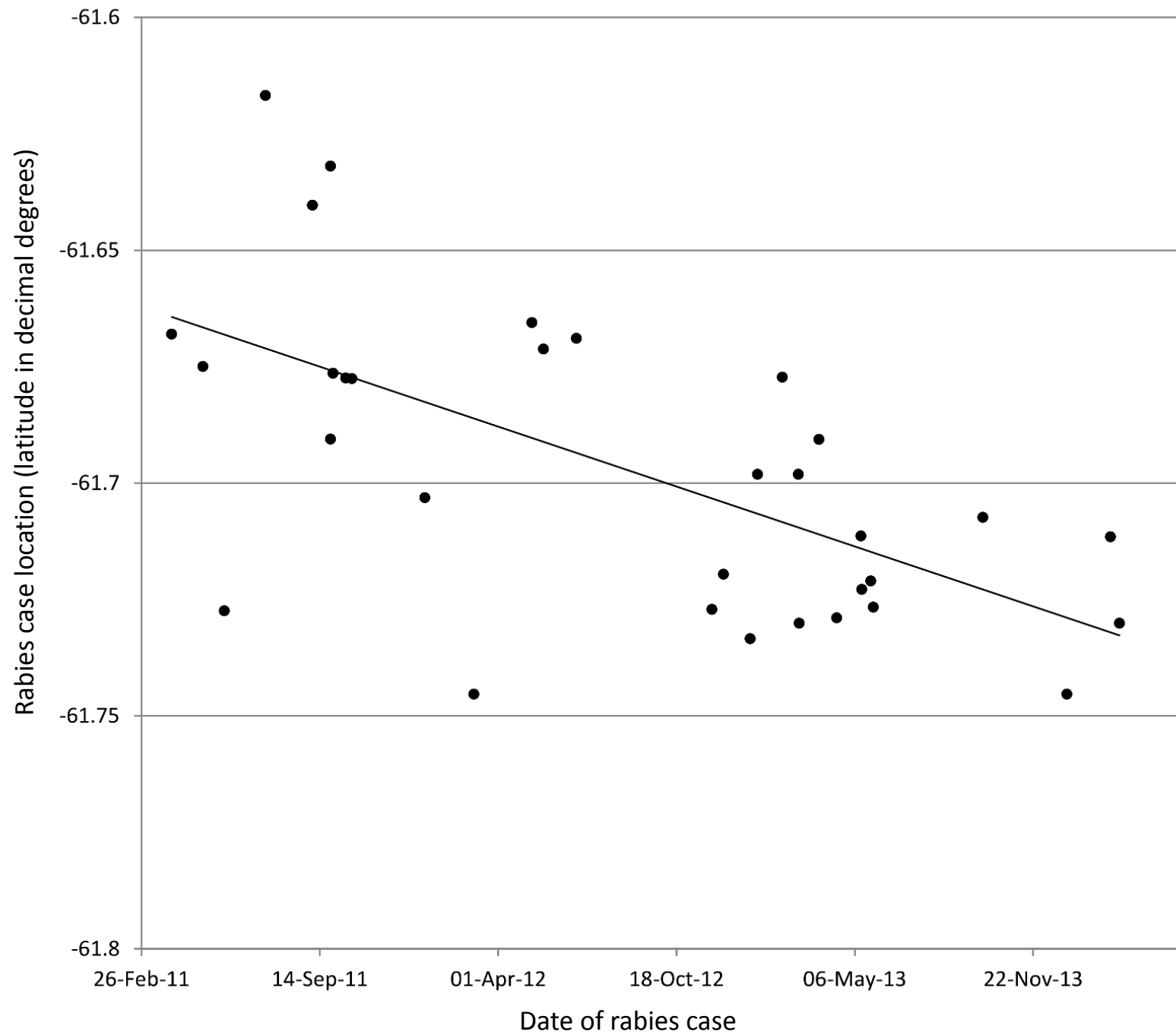

Supplement: Figure S2 — Relationship between latitude and date of submission of rabies cases. There is a significant correlation between latitude of case origin (in decimal degrees) and date of case submission (Pearson's correlation coefficient of −0.63 [95%CI −0.80 to −0.35, p = 0.0001]). These data suggest a temporo-spatial spread from North to South. (PDF) [file pntd.0003251.s002.pdf]

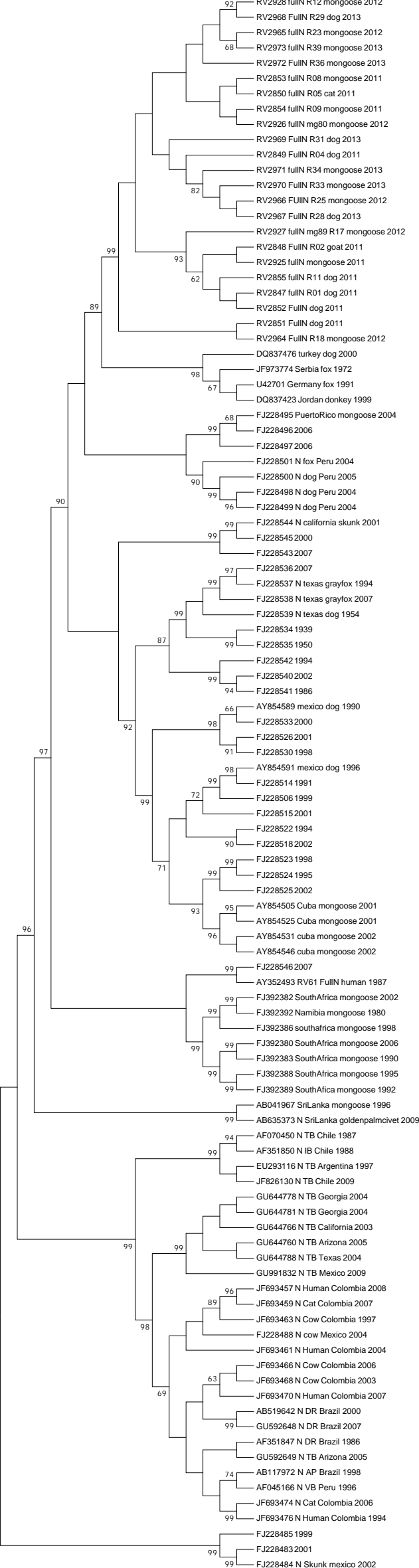

Supplement: Figure S3 — Maximum likelihood phylogenetic tree, comparing the same 1350 bp N-gene sequences as in Figures 3 and S1 using the same TN93 nucleotide substitution model with rate variation among sites and a proportion of invariant sites. Bootstrap values (percentage of 100 replicates) are given at significant nodes. (PDF) [file pntd.0003251.s003.pdf]
